# Hostage negotiator resilience: A phenomenological study of awe

**DOI:** 10.3389/fpsyg.2023.1122447

**Published:** 2023-04-11

**Authors:** Jeff Thompson, Elizabeth Jensen

**Affiliations:** ^1^Columbia University Irving Medical Center, New York, NY, United States; ^2^Institute for Conflict Management, College of Leadership and Public Policy, Lipscomb University, Nashville, TN, United States; ^3^Clinical Psychology, Long Island University-Brooklyn, Brooklyn, NY, United States

**Keywords:** hostage (crisis) negotiator, awe, resilience, hostage and crisis negotiation, phenomenology

## Abstract

Law enforcement crisis and hostage negotiators (CHNs) are tasked with resolving incidents that are stressful, unpredictable, and often dangerous. These negotiators must work as a team and be able to successfully utilize a variety of skills in order to gain the subject’s voluntary compliance and peaceful surrender. It is necessary for negotiators to continually practice these skills while also, and importantly, care for their own wellbeing. This study examines how a phenomenon, awe, when viewed as a resilience practice, can support law enforcement crisis hostage negotiators with their crisis work and personal wellness. Utilizing phenomenological methodologies, the findings demonstrate that reflecting on awe experiences had an overall positive impact on the negotiators in both their professional and personal lives. Based on the results, it is suggested that awe practices could be incorporated into future negotiator trainings in order to increase resilience and assist negotiators personally and professionally.

## Introduction

Law enforcement can be stressful, unpredictable, and dangerous. This is especially the case for crisis and hostage negotiators (CHNs) who are involved in tense situations and often interact with people who are in a mental health crisis. In order to remain effective in saving lives, it is necessary for these elite members of law enforcement to continually train together in order to successfully work together as a team and efficiently utilize the requisite skills. Emerging research has been examining how awe can be viewed as both a resilience practice while also being supportive of other practices such as cognitive reappraisal, finding meaning and purpose in life, gratitude, optimism, self-compassion, self-efficacy, and social connectedness (Tabibnia, 2020; Thompson et al., 2022; Thompson, 2022a). Additionally, when viewing awe as a resilience practice, it has been suggested as having the potential to enhance the wellbeing of these negotiators while also contributing to their efficiency in crises (Thompson et al., 2022). This exploratory study is a phenomenological analysis of law enforcement CHNs engaging in a specific resilience practice: reflecting on awe experiences. This paper begins by providing an overview of the crisis negotiator’s role and responsibilities, followed by an exploration of how awe can serve as a potential resilience practice and support negotiators. Next, the study’s methodology, results, and findings are provided. Lastly, the conclusion offers suggestions for future research examining awe, resilience, and hostage negotiators.

## Crisis hostage negotiator

Crisis and hostage negotiators (CHNs) are specialized, law enforcement officers who emerged in the early 1970s (McMains and Lanceley, 2003; Grubb et al., 2022), prompted by a terrorist-related hostage-taking incident at the Munich Olympics (Johnson et al., 2018). Since then, negotiators have been trained to utilize crisis intervention techniques in high-conflict and life-threatening situations in order to resolve challenging and stressful incidents (McMains and Lanceley, 2003; Johnson et al., 2018; Grubb et al., 2022; Thompson et al., 2022). CHNs interact with people who are often in some form of a crisis and as such, the CHN attempts to defuse the frustrations and tensions within the situation. According to Hoff (1989), a crisis can be defined as any event that threatens to overcome the individual’s means to cope. This perceived conflict is viewed as insurmountable by the person involved in the crisis and the need to engage in unusually extreme methods becomes a viable option (Johnson et al., 2018). For instance, a person may resort to taking hostages to solve their problem, another person may stand at a bridge and threaten death by suicide, or someone else may threaten to harm another person or entity through the use of violence (Johnson et al., 2018). These crisis events typically stem from a variety of traumas, but most involve the significant loss of a relationship, rejection, termination from employment, financial loss, decreased health, or the impending loss of freedom (Johnson et al., 2018).

A major component of the CHN’s strategy is to slow down the crisis incident in order to allow time for the person to vent their feelings (i.e., anger, frustration, sadness) and consider their current and future actions (Van Hasselt et al., 2008). In order to slow this process down, CHNs use skills which consists of active listening, empathy, rapport, and behavioral influence (Van Hasselt et al., 2008). Negotiators are also trained in a host of other areas to successfully navigate these highly stressful situations, such as, but not limited to stress management, mindfulness, working with victims and families, and other aspects of mental health trainings (Mullins, 2002). Research by Grubb et al. (2019) have found that certain characteristics were viewed to be important qualities for a negotiator to possess in order to be successful with negotiations (i.e., empathic, demonstrating respect for another person, flexibility, law enforcement experience, patience, resiliency, compassionate, trustworthy, mental stamina, and insightfulness). As such, the negotiators’ primary goal is to obtain the person’s cooperation and compliance without the additional use of the tactical/operations team (Johnson et al., 2018).^1^

## Awe

Various definitions of awe have been provided by researchers and generally, they involve two critical elements: vastness and a need for accommodation (Keltner and Haidt, 2003). Vastness can refer to something both physical as well as conceptual, such as time or power (Chirico and Gaggioli, 2021; Cuzzolino, 2021; Thompson, 2022a,b). As a result of this vastness, it requires a new mental schema to be constructed, which has been referred to as a need for accommodation. This accommodation is something that challenges our mental representation, due to the vastness and poses difficulty in assimilating the novel experience into the preexisting mental schemas (Keltner and Haidt, 2003; Dong and Ni, 2020; Thompson, 2022a,b). Emerging awe research has refined the definition of awe as being a complex emotion that is experienced in response to something, or someone, extraordinary and challenges the person’s current thinking (Stellar, 2021; Thompson et al., 2022). Awe is primarily a positive emotion, however, it can include negative elements such as fear or horror (Chirico and Yaden, 2018; Guan et al., 2019). When awe is considered in the context of a positive emotion, it can lead to an increase in the person’s ability for attention (Chirico et al., 2017), thought formulation, and can broaden one’s “physical, intellectual, and social resources” (Dong and Ni, 2020, p. 905). Furthermore, positive awe can elicit greater feelings of calmness and personal control of a situation (Guan et al., 2019). When viewed in the context of negative awe experiences, this can be associated with feelings of powerlessness, a decrease in self-control, and uncertainty (Guan et al., 2019).

Awe can be experienced in a variety of settings including nature, space, music and the arts, spiritual and religious moments, interpersonal relationships, and through one’s accomplishments as well as that of others (Shiota et al., 2007; Yaden et al., 2019; Sturm et al., 2020; Graziosi and Yaden, 2019; Thompson et al., 2022). Awe can be elicited through direct, in-person experiences, while also through sharing and reading narratives, photos, video, audio, and both augmented and virtual reality (VR) (Chirico et al., 2017; Thompson, 2022a). This study focuses on the positive attributes of experiencing awe. Previous research has shown that awe can increase overall wellbeing (Graziosi and Yaden, 2019), contribute to feeling connectedness with others (Chirico et al., 2017) (and nature), critical and creative thinking, curiosity, decision making, “feeling small” (Nelson-Coffey et al., 2019, p. 2) in a positive way, focus, gratitude, humility, open-mindedness, optimism, tolerating ambiguity, and handling uncertainty (for a review, see Allen, 2018; Cuzzolino, 2021; Thompson et al., 2022).

Awe has been described as both an epistemic emotion and a self-transcendent experience (Chirico and Yaden, 2018; Thompson, 2022c). As an epistemic emotion, experiencing awe can reveal gaps in knowledge while then motivating the person experiencing awe to want to fill those knowledge gaps. When referenced as a self-transcendent experience (STE), awe can allow the person experiencing it to look beyond themselves and contribute to prosocial behaviors (Guan et al., 2019; Koh et al., 2019; Cuzzolino, 2021; Thompson, 2022c). Studies have shown examples of prosocial behaviors including generosity, donating, volunteering, and compassionate action for both others and the environment (Guan et al., 2019). Additionally, and with direct relevance to this study, reading and sharing narratives have been examined as reflective, wellness, and an enhancing resilience practice for evoking awe in others (Rudd et al., 2012; Piff et al., 2015; Walker and Gilovich, 2021; Cuzzolino, 2021; Thompson, 2022c).

## Resilience

Previous research has suggested that experiencing awe and other related resilience practices can support both a CHN’s wellbeing and enhance their abilities related to their work in crisis incidents (Thompson et al., 2022). Much like awe, there are various definitions of resilience. Adapting previous versions from researchers (Southwick and Charney, 2018; APA, 2020; Thompson et al., 2022), the authors define resilience as a collection of practices to support an individual’s wellbeing that can be utilized proactively, in the midst of a crisis or stressful moment, and as part of their recovery. For the purpose of this study, examples of evidence-based resilience practices include cognitive reappraisal (Hanson, 2018; Southwick and Charney, 2018), connectedness (Suttie, 2017; Nitschke et al., 2021), empathy (Waddimba et al., 2021; Wu et al., 2022), gratitude (Emmons, 2010; Millstein et al., 2016), meaning and purpose in life (Aten, 2021; Ballard and Ozer, 2016) mindfulness (Kwak et al., 2019; Antonini Philippe et al., 2021), optimism and prospection (Reivich and Shatte, 2003; Bulley and Irish, 2018), and self-efficacy (Maddux, 2005; Bandura, 2008). Recent, exploratory research conducted by this author has established a relationship between these specific resilience practices with awe (Thompson, 2022a,b,c, 2023a,b). Those studies have shown that experiencing and reflecting on awe can serve as a gateway to other resilience practices and support their overall wellbeing. This study attempts to advance the findings from the aforementioned awe-resilience studies by directly engaging CHNs to discern the potential role of awe in relation to their work.

## Methodology

Phenomenology, a qualitative research methodology, was determined to be the most suitable approach to conduct this study. Phenomenology explores how a phenomenon is experienced by an individual. The phenomenon being examined for this study is awe. In addition to phenomenology, interpretative phenomenological analysis (IPA) supported this study’s overall methodological approach. IPA is concerned with small, homogenous sample sizes that can provide rich data with an emphasis on the quality of the data and not the quantity or necessitating a larger sample size (Smith et al., 2009; Frechette et al., 2020).

The research question guiding this study was: How do law enforcement hostage negotiators experience awe and how does it, and related resilience practices, impact them (professionally and personally)? Nine negotiators participated in the study which is a sample size consistent with both phenomenology and IPA (Polkinghorne, 1989; Groenewald, 2004; Smith and Nizza, 2021). Five negotiators were from the United States, two from New Zealand, and one from Australia, Canada, and the United Kingdom. They were invited *via* email to participate in the study through prior, established professional relationships either directly or through professional peers. A balance between gender was intended, as well as obtaining diversity *via* different geographic locations. In order to qualify for participation, each person had to be an active member of law enforcement and currently on their agency’s crisis/hostage negotiator team (CHNT). Each participant had to have been involved in at least five incidents, where they served as the lead negotiator. This requirement was necessary for them to be able to reflect on multiple CHNT experiences concerning awe. As displayed in Table 1, each participant was highly experienced in crisis incidents. Table 1 details additional demographic data of the participants.

**Table 1:** Participant demographics.

	Age	Years of law enforcement experience	Years of CHNT experience	Number of CHNT incidents	Number of CHNT incidents as the lead negotiator
Female 1	44	23	12	500	240
Female 2	39	15	12	300	50
Female 3	53	28	15	200	50
Female 4	37	15	11	65	20
Male 1	44	19.5	10	50	20
Male 2	55	21	16	250	50
Male 3	49	26	12	400	200
Male 4	49	26	17	125	25
Male 5	42	11	12	66	50
*Mean*	*48.44*	*23.94*	*12.88*	*215.55*	*74.11*
*SD Standard Deviation*	*8.88*	*8.21*	*2.47*	*161.69*	*85.88*

Consistent with qualitative research methodology, specifically phenomenology and IPA (Smith and Osborn, 2003; Smith and Nizza, 2021), semi-structured interviews were conducted with each participant separately to gain their unique perspective of the phenomenon, awe. Additionally, researcher-specific questions were utilized to elicit how awe has impacted their work as a law enforcement CHN. During the interview, other related, positive emotions and resilience practices were also examined. All participants completed an electronic consent form prior to their interview. The study was approved through the first author’s institutional review board.

The data analysis and theme development follow the suggested practices for conducting general phenomenological studies and specifically interpretative phenomenological analysis. Previous qualitative research conducted by the first author helped guide the development and analysis of the data (Thompson, 2022a,c, 2023a,b). This included Thompson et al. (2022) establishing the relationship between resilience skills, and specifically experiencing awe, with CHN’s personal resilience and qualities of negotiator effectiveness. IPA was determined to be the most suitable methodological approach as it seeks to first, make meaning of the experiences of the individual related to the phenomenon of awe, and then shifts to the researcher’s ability to determine themes derived from multiple participant’s experiences (Smith and Osborn, 2003).

Each interview was conducted *via* Zoom and lasted for approximately 50 min. The questions for the semi-structured interviews were adapted from previous, similar awe research (Cuzzolino, 2021) and informed more broadly by other awe and resilience studies (for example, see Thompson et al., 2022). There were 19 questions to guide the interviews (see Appendix A), however, they were not asked in a specific order and they were not all asked to each participant.

The interviews were recorded and then the deidentified audio files were transcribed by a third-party service. The first author initially analyzed each interview separately and then again collectively in order for themes to emerge (van Manen, 1990; Smith and Osborn, 2003; Creswell, 2007; Bonner and Friedman, 2011; Smith and Nizza, 2021). Again, consistent with IPA, the first author’s understanding of the phenomenon and notes contributed to the emergence of themes (Smith et al., 2009). The first author has conducted previous, similar research on the phenomenon of awe (2022) as well as with hostage negotiators. Further, he is a retired law enforcement detective having served for 20 years including in the role of a hostage negotiator and trainer.

The related research conducted by Thompson (2022a,b,c) and colleagues (2022) offered specific guidance on the establishment of themes related to awe and resilience practices. The emerging themes were compiled into a database where each participant was given a deidentified label (for example M1 for the first male interviewed and F1 for the first female interviewed). The second author’s role in this process was reviewing the individual interview transcripts and notes, the group themes, and then providing edits, questions, clarifications, and overall feedback. In instances where there were questions and suggested additions or dissimilarities, the authors had further discussions to reach a consensus. The second author’s background includes experience in crisis-related incidents, prior mental health training, and education in various fields of psychology (i.e., clinical, counseling, and forensic).

As previously discussed, the existing literature has demonstrated a variety of methods of eliciting awe, including through sharing narratives and reading the experiences of others. To further examine the potential that sharing a narrative can have with awe, and overall wellbeing, this study advanced the recommendations provided by Cuzzolino (2021) and Thompson (2022a,b). A post-interview survey was sent to the participants’ emails to gauge the participants’ perspectives about being interviewed.

Finally, the findings, interpretations, and themes are presented jointly and supported by excerpts from the participants. This approach is consistent with previous resilience research (Thompson, 2020; Thompson and Drew, 2020) and specifically, awe (Thompson, 2022a). IPA research has been described as purposely avoiding rigid and specific structuring across studies, as it is not a descriptive methodology (Smith and Nizza, 2021). Instead, the IPA principles are utilized based on the needs of the researchers as they explore a phenomenon (Smith and Osborn, 2003; Smith et al., 2009; Smith and Nizza, 2021).

## Results and Discussion

During the interviews, participants explained how awe and related resilience skills contributed to their effectiveness in their crisis work, as well as their personal wellbeing. As discussed in the previous section, through IPA, 15 themes emerged. This number of themes is consistent with previous, similar awe research conducted by the first author including the 18 themes that emerged from the study on The Awe Project (Thompson, 2022a) which guided the theme development for this study. Importantly, the themes from The Awe Project study were not pre-selected as themes for this study, instead, through the analysis, some similar and different themes emerged.

These themes are alphabetically displayed in Table 2 and are further discussed throughout this section to demonstrate the complex nature of awe and specifically, the interconnectivity between the various attributes of awe and resilience as described by the participants. An example of this complexity and interconnectedness is the theme of “accomplishments.” Reflecting on accomplishments has been previously explained as a category of evoking awe. However, it can also enhance resilience as reflecting on personal accomplishments can additionally evoke gratitude and supporting self-efficacy – both of which are also related to experiencing awe. This example demonstrates how awe can serve as a gateway to other resilience practices and support overall wellbeing.

**Table 2:** Awe and negotiator-related themes.

Theme	Example
Accomplishments	*[Being a CHNT is] just one of those things, you are really proud of it.*
Cognitive reappraisal	*It just kind of changes your outlook on life and your lifestyle.*
Connectedness(Teamwork)(Personal relationships)(Mentors)	*There’s no way you can do it without partners* *It has not been easy, I’ll be honest, but we have [them along with their spouse] managed to get through* *You have to find that mentor to kind of guide you*
Curiosity	*I honestly think from my experiences and talking to people that curiosity helps negotiators be genuine. Because otherwise it could turn into trickery.*
Empathy	*It’s seeing the world through their eyes, which is the most important thing.*
Gratitude	*I would describe it as a privilege as well, working amongst a whole lot of people who were all striving to achieve the same goal really.*
Humility	*It is so important to have that team atmosphere and put all egos aside.*
Learning/filling knowledge gaps	*You want to know what makes them happy. What drives them? What keeps them going?*
Mindfulness	*Being able to separate yourself from whatever else is going on in your life… having the ability to be able to put that aside and just focus solely on the negotiation is quite important.*
Open-mindedness	*You have to be able to consider things in a very broad way.*
Prospection and optimism	*I think that becomes, again, overwhelming, so just keeping that spirit of hope is really important.*
Self-care	*I think finding the ability to prioritize self-care, has been a really important aspect for me personally.*
Self-efficacy	*I’ve found that you have to be your best advocate yourself. You need support, but it ultimately lies on your shoulders.*
Self-transcendent experience	*You were part of something that was greater than you.*
Uncertainty and ambiguity	*I think negotiators really have to be comfortable with the uncomfortable.*

Themes listed alphabetically.

### Negotiator skills

Prior to examining the direct role awe can have in their crisis work and personal lives, the qualities of effective crisis and hostage negotiator (henceforth referred to as negotiator) were explored first. Consistent with the literature (Johnson et al., 2018; Grubb et al., 2019; Thompson et al., 2022), multiple participants reflected on how important it is to demonstrate empathy, in order to be an effective negotiator:

Empathy is number one, for sure. If you’re not empathetic, you’re just not going to be able to connect with somebody that’s going through a crisis.

The ability to develop empathy and that emotional intelligence to pick up on not just the black and white body language, but to feel the emotion coming behind people’s words.

That they have empathy and they listen. Everyone always thinks that to be a good hostage negotiator, you have to be a good talker. And it’s not that at all, you have to be able to empathize with that person, with what they’re going through, in that specific moment or situation.

As previously examined and demonstrated by Thompson (2022a,b) and Thompson et al. (2022), experiencing and reflecting on awe can enhance empathy. Additionally, and related to awe, open-mindedness as well as being able to efficiently handle uncertainty were described by negotiators as being necessary as well:

You have to be able to consider things in a very broad way because ultimately you don’t know where the conversation is going to go, and so if you’re really narrow-minded with your line of questioning for those individuals that you were reaching out to for information, that can be a problem.

Participants also reflected on recognizing the seriousness of their work and the pressure involved with regard to their ability in being effective:

To me, you have to be invested and you have to have the passion to do this, and really understand it and the responsibilities that come with it.

If I get it wrong, that person’s going to die. That’s my grounding, that’s my reality. Whether there’s a bit of me that thinks this is an attention seeker, this is just a grab of my time. I tell myself, ‘If I get this wrong, they’re going to die.’ And that’s how I [keep myself in check].’

We’re going into high pressure. What does HNT respond to? Crisis. It’s a crisis every time. So, the pressure is on. And every word that you say may determine the outcome of that.

CHNT literature and trainings have well-established the significant role active listening has in negotiators being effective.^2^ The following two participants provided insight into why it is so important, including how it can fill a social void for the subject:

The reality is that it’s really about listening. Listening to that person, because that’s what they’re lacking. They’re lacking something, or someone, that understands them.

You’re going out there and you’re trying to be there for someone who hasn’t had anyone to listen to them, or they didn’t feel like they can reach out and have that support. It’s an important job. It’s exhausting. It takes a lot out of the individual. But it is an important task.

The above participant also explained how tiresome it can be for the negotiator when using the necessary skills properly. The following participant added to the challenges of being a negotiator and how it requires self-control, patience, and focus:

Just being able to keep calm I think, and keeping control of your voice, control your emotions as well. Being able to separate yourself from whatever else is going on in your life … having the ability to be able to put that aside and just focus solely on the negotiation is quite important.

As part of negotiator effectiveness, the importance of operating as a team was stressed as being vital to a negotiator’s success by many participants.

I think it’s less about you being super smooth with your words and more about being a good team player … there’s no way you can do it without partners that are on the same page, understanding what you need.

The following participants expanded on the value of having a cohesive team environment by explaining how an incident is too much for one person to handle. The other team members can offer the lead negotiator valuable assistance:

Oh my God, I think [the team concept is] hugely important, because I’ve certainly had moments in a negotiation where you can’t. It’s difficult to get that connection and you are like, ‘Oh goodness, I’m all out. I just don’t know what to do from here. So, it’s so good having someone else there who can be like, ‘Hey, why don’t you look at this angle?’ … Someone else on my team might have this really awesome idea that I haven’t even thought about. And you can miss those opportunities if you don’t work as a team.”

You have to be ready for anything and that’s why you need a good team around you. Negotiation is a team sport because the guy might throw something out there. You have no idea what he’s talking about but you don’t have time to go research it or go look it up. That’s when you need your teammates.

This participant further demonstrated the importance of working as a team by relating it to humility and being goal-centered:

It is so important to have that team atmosphere and put all egos aside and really work as one. Yes, you are a team, the mission is the same.

Previous research has investigated the benefits of a person possessing curiosity and suggests that those who experience awe are “highly curious and motivated to learn about the world in which we live” (Anderson et al., 2020, p. 762). Further, Anderson et al. (2020) surmise that awe “directs attention away from the self, outwards to one’s physical and social environment, a precursor to curiosity” (p. 764). It has also been suggested that curiosity can support police leaders in addressing modern issues they are facing and how experiencing awe can contribute to developing meaningful strategies to address those concerns and issues (Thompson, 2022b). Studies have demonstrated that experiencing awe can result in an increased curiosity for participants (McPhetres, 2019; Anderson et al., 2020; Thompson et al., 2022; Thompson, 2022a) and therefore curiosity was discussed with participants to examine its value for negotiators. This participant explained the significance of curiosity with respect to the work of negotiators:

I’m real curious. I’m real curious to know about that person. What makes them tick? I think that for a successful negotiation you want to know what makes them happy. What drives them? What keeps them going? So, curiosity is huge. I’m real curious.

The following negotiator established a connection between curiosity, handling uncertainty, being uncomfortable, and how it is something negotiators can enhance:

I think negotiators really have to be comfortable with the uncomfortable … I think if you’re curious, you just might have some ability to connect with people that you typically wouldn’t maybe interact with … I think if active listening is a trainable thing, I think being curious is probably equally so.

The following participant clarified what makes a negotiator effective while connecting curiosity with empathy:

Curiosity starts by you just listening. Oftentimes, people think to be a good negotiator you have to be a good talker. And I say, no, you have to really listen because they haven’t had anybody listening to them for many years… Stay quiet, listen. Take those moments and be curious. And that’s when you realize, oh, this is what’s going on. Now, utilizing the information you got and really trying to connect with that individual by seeing the world through their eyes. And I always say it doesn’t mean that you have to agree with them, but it’s seeing the world through their eyes, which is the most important thing.

Another participant further contributed to emphasizing the necessity of curiosity, as it allows a negotiator to gain a more accurate perspective of the subject’s situation:

… wanting to be curious enough to find out what their real story, because what they tell you first, probably isn’t going to be the real story, and you’ve got to be curious and gentle enough to get the story out rather than be a cop and try and force the story out… the curiosity is about unpicking their story gently so that they don’t feel as though they’re under any pressure. They don’t feel as though they are being interrogated by a cop. They are in a position where they actually want to talk to you, and we have to give them that position where they feel comfortable that they want to talk to us.”

The above quote importantly shows how the negotiator uses curiosity to create a trusting and rapport-building environment that is not adversarial but instead, one where the subject will want to willingly share information. In order to build rapport and trust with the subject, and create an environment for them to share more information, a negotiator must be genuine when using their skills:

I honestly think from my experiences and talking to people that curiosity helps negotiators be genuine. Because otherwise it could turn into trickery.

The following participant shared how their curiosity is part of their overall personality and not limited to their negotiation work:

I’m very curious about people and I’ll strike up a conversation with a stranger just because I think it’s interesting to see how different people live life and what they’re all about.

In addition to curiosity, creativity is another important skill for negotiators to possess, due to the constant uncertainty they are faced with (Zhang et al., 2021):

Not every negotiation is the same. We do teach the negotiation course and we give you procedures. And procedures are guidelines… but that doesn’t mean that you box yourself. You always have to be creative… I’m not saying you have to be a trickster. You have to be able to think outside the box. There’re always gray areas. You can’t say, “Oh, never say no.” And being humble, it’s just you can’t have an ego. You can’t. This job, you’re going into a situation when someone is going to kill themselves. They are at the end of their rope, and there's no hope. And you're standing there, and you cannot put your needs, your ego, first. You have to be very humble. You have to be very understanding. And again, being empathetic.

Self-efficacy, or believing in one’s own abilities, has been described as an important quality for negotiators (Thompson et al., 2022). In part, as the following participants explained, it helps develop confidence and avoids second-guessing oneself.

I’ve found that you have to be your best advocate for yourself. You need support, but it ultimately lies on your shoulders.

I think it’s super cool that you have this opportunity to be on the phone potentially with somebody who has other plans or other ideas, and maybe you’re going to persuade them otherwise, and that is very, very neat.

Nobody wants you around till they need you and then when they need you, they need you bad. When you walk in and a lot of it’s just having confidence in your own abilities. … It feels pretty cool.

The above comments also revealed an element of self-efficacy that included reflecting on what they do for a living and enjoying it. Participants were asked what impact reflecting on their role as a law enforcement crisis hostage negotiator have:

And I’ve worked a lot of different assignments with the [police agency], from narcotics, to internal affairs, to patrol, to training. And the hostage negotiation part of it is my favorite part of my profession.

It’s good. I love it. And you mentioned curiosity before, and I think that’s really what keeps me going, is I like to know what’s going on, and I like to be involved and stuff like that.

It’s just one of those things, you’re really proud of it.

Finally, and previously mentioned, the above reflection shows the interconnection between many of the negotiator skills with each other and with the attributes of awe. In this case, it is curiosity, accomplishments, and gratitude.

### Defining and understanding awe

Guided by the existing research that has already established the relationship between awe, wellness, and resilience (Tabibnia, 2020; Thompson, 2022a), the focus of the interviews shifted more directly to the research question and specifically, the phenomenon of awe. The participants were asked to define awe in their own words, and this negotiator related it to beauty:

Awe is something that is a beautiful thing, it’s something that brings you to say “wow” or to feel amazed by some of these things that you’re experiencing. I think that awe can sometimes also be uncertainty.

Importantly, previous research has demonstrated handling uncertainty as being necessary for negotiators’ effectiveness (Thompson et al., 2022). Further research has shown experiencing awe can support an individual’s ability to handle ambiguity and uncertainty (Thompson et al., 2022).

According to this participant, awe can be experienced in the company of others and felt when admiring someone else’s accomplishments in a positive manner:

I’m impressed … I think it’s the opposite of jealousy.

The above negotiator’s statement demonstrates that when experiencing awe, it can contribute to humility (Stellar et al., 2018) and negotiator effectiveness. The following participant’s definition details the complexity of awe which involves additional emotions as well:

That almost step back of whoa, in a positive sense. Not in a, ‘Oh my God,’ but a, ‘Oh wow.’ Yeah, that would be my definition of awe … other emotions associated directly with awe: happiness, euphoria, surprise, respect, adulation, generally those positive kind of emotions.

Finally, numerous participants explained the intensity related to awe experiences which can also result in them not being able to fully describe it. This is consistent with Keltner and Haidt’s (2003) seminal work on awe and how when it is experienced, it elicits awe as a need for accommodation. This refers to the person experiencing awe not initially being able to comprehend or grasp what is occurring and therefore has to develop a new mental schema to generate an understanding:

Almost like overwhelming, or disbelief, or something that blows your mind. It can cause you to get a bit emotional … overwhelming joy.

It’s great. And you’re in awe of that, in shock … happiness. Sometimes, you’re in awe that you can’t figure this out because it’s confusion. [The emotions are] intense … It’s obviously something that’s out of the norm.

It’s one of those things that you almost literally don’t have words to describe it.

The definitions provided by these negotiators are consistent with existing literature that has defined awe as being: complex, intense, not easy to explain, and mainly a positive experience (Dong and Ni, 2020; Thompson, 2022a).

### Awe stories

Participants shared moments related to their negotiation work where they experienced awe. This included reflection on the seriousness of these crisis incidents, including the life-or-death consequences that are part of it. Much like general experiences of awe, the following participant shared how this can lead to intense emotions:

The first negotiation that I went to where I actually thought this person was going to jump. And it really struck me like, ‘Wow, I could be looking at this person and then not looking at this person.’ There was this huge sense of overwhelming emotions.

The participant’s reflection continued by realizing how important it is to manage one’s emotions. If emotional responses are not managed properly, they could be detrimentally contagious for the other person:

It’s really just about taking a breath. If I become rushed, I come across as stressed or panicky, then that is almost certainly going to have an effect on that person.

The participant continued by explaining how goal-setting and controlled breathing restored them to feeling calm, focused, and able to remain present and in the moment:

So, it’s really just about knowing what my goals are, knowing what I’m there to do, what I’m there to achieve, and then how I’m going to do that. So just taking that minute to just breathe and think, ‘What is it that I need to do now? What’s important right now? What’s…’ And then working towards achieving an end goal.

Finally, the participant concluded how a vital practice in resilience and positive psychology is having a sense of gratitude and savoring the moment when it ends well:

When it’s a happy ending you get all those happy, good vibes and you’re like, ‘I just saved a life.’

This type of profound reflection that the negotiator had with regard to saving another person’s life and savoring the moment was expressed with greater intensity by this participant:

This guy’s going to live now. He wants to live. He wants to get help. And possibly something I’ve said has helped him unravel 44 years of shit and he’s prepared to try and do something about it now. And that was my awe – that, ‘Christ, wow.’

Not all of the work-related awe stories shared by participants were positive experiences. The literature has shown that although awe is often related to positive moments, it can also be associated with negative emotions and non-pleasant situations (Gordon et al., 2017; Guan et al., 2019):

I did have a bit of awe when I dealt with a guy who had his two daughters as hostages in his house, but it wasn’t awe in a good way ….

He had his two daughters at knife point and held them for over 12 hours in a room upstairs in his house. He refused to come out. I tried as much as I could to negotiate, to get him to come out.

I tried a number of different techniques and although he did speak with me and yell at me, which I took as a positive thing. He refused to come out and the tactical team ended up having to go in to get them out once they worked out what their tactics would be. So, I guess I was in awe of that really. It was a big day.

I would describe it as a privilege as well, working amongst a whole lot of people who were all striving to achieve the same goal really. And I had an opportunity to be able to use my skills in order to try and get him to come out. Unfortunately, it didn’t work.

Although the above story was described as a negative experience, it can also be viewed in terms of cognitive reappraisal that contained positive elements as well. Although the negotiation did not gain voluntary compliance from the subject, the overall incident was peacefully resolved. The negotiator’s actions contributed to the tactical team’s success by “stalling for time” (Noesner, 2010; Thompson, 2014), which refers to a well-established negotiation tactic of using the concept of time and patience within the negotiation strategy. In this case, the negotiator’s patience allowed time for the tactical team to develop, and subsequently, implement their plan.

Furthermore, self-compassion was evident. As described in the literature (Neff, 2011), self-compassion involves acknowledging things will not always go as planned and be positive despite our best efforts. This is further linked to another resilience attribute, possessing self-efficacy, or believing in one’s abilities. Both self-efficacy and self-compassion allows the resilient person to “bounce back” when a situation does not go well or as planned.

Additionally, another positive attribute the participant reflected on from this experience was the sense of connectedness and gratitude. All of the police personnel on the scene, from various units, were working together in one unified goal – saving people’s lives. This reflection allowed the participant to sense awe, as well as other related resilience attributes such as gratitude, connectedness, self-compassion, and self-efficacy.

### Personal awe moments

In addition to sharing work-related moments of awe, participants were asked to share personal, non-work related stories where they experienced awe. This question was purposely asked in relation to finding meaning and purpose in life (M/PiL). Meaning in life can be described as the extent to which a person believes their life has a purpose and is significant (Rivera et al., 2020). As such, being a CHN and involved in crisis incidents can contribute to an overall M/PiL, however it is also important for law enforcement members (as well as the general public) to not be enmeshed in their work and develop an unhealthy attachment.

Consistent with previous research involving awe stories being shared (Thompson, 2022), participants in this study reflected on moments involving those close to them. This included profound moments of giving birth:

“You think, ‘This is going to be awesome. I can’t wait to have a child.’ And then the minute they’re here, you’re like, ‘Oh my gosh, I am so responsible right now for the outcome of this little tiny being.’

And then all these things that looking back came up the minute they were born that you have to decide for this little tiny being is very scary. And if you don’t have hope that things are going to be okay and that you’re going to do the best you can, I think that becomes, again, overwhelming, so just keeping that spirit of hope is really important.”

The above participant details another important element related to awe and resilience in general: hope and optimism (Thompson et al., 2022). Having hope and optimism refers to an individual believing positive change is possible and they can achieve realistic goals they have created for themselves (Reivich and Shatte, 2003; Southwick and Charney, 2018).

Personal accomplishments, as well as sensing awe in those close to them (Graziosi and Yaden, 2019), were both previously established elicitors of awe (Walker and Gilovich, 2021; Thompson, 2022c). The following participant reflected on not just their own financial success but how it is intensified because it was achieved along with their spouse:

That we are doing quite well now financially with properties that we bought … and I’m in awe of the fact that we’ve managed to achieve a goal that we had set years ago, which is pretty cool. I’m pretty happy about that”

Finally, another common elicitor of awe is nature. This participant reflected on a recent walk in nature and how it captivated them:

Walking the dog about two days ago. It was a Spring morning and it was cold. The sun was shining beautifully; the mist was rising off the lake. The birds were singing the morning chorus. There was no one about, and I was walking with the dog and that was an awe moment. That was a real, ‘Wow. Yeah. Bloody hell.’ That feeling of just peace and calm, just for that second or two when I was just able to realize what wasn’t around me. That was definitely [awe].

The above story showed that in addition to nature being able to elicit awe, it is also those ordinary, everyday moments that can also elicit awe when approached with an open-minded perspective (Schneider, 2009; Graziosi and Yaden, 2019). This demonstrates how in terms of well-being, enhancing resilience is not necessarily about changing the conditions around us, but instead, it involves possessing a resilient perspective of what is occurring in our environment.

Consistent with the literature, participants reflected on how awe can invoke feelings that they are a part of something larger than themselves, especially in relation to their crisis work:

It doesn’t take one person to resolve a crisis. And when you are able to take someone’s hand and walk them over to the other side, the good side of the bridge, and you look around you, all the people that were there, and the support that they gave you, most of the time in very, very, bad elements, cold, hot, hungry, and you’re doing your best to save a life, and they hold you up. They’re there for you. You know that if something happens, they will step in. When you look around you and you realize that there’s so many good people that are doing a great job, it’s very hard to explain, but it’s like, you know what? Everything else is just a speck. It’s just dust. You built those bonds with people that are never broken… you were part of something that was greater than you.

This sense of connectedness to something larger than themselves is a positive result of experiencing awe, as it can motive people to support others. This is a prime reason awe has been described as a self-transcendent emotion:

You become, to some extent, connected, or emotionally you feel a sense of responsibility for your role as a negotiator. You can’t just be like, ‘Oh my God, okay. It’s an hour, I’ve got to go home.’ It’s not like that. You become part of something bigger than yourself.

Experiencing awe has also been described as creating a perspective shift in those who experience it:

[Awe] just kind of changes your outlook on life and your lifestyle. Just appreciate things more.

The above negotiator reflection shows how a perspective shift can also evoke gratitude.

### Additional awe themes

Although part of a negotiator’s success lies in their ability to believe in their abilities, participants were also prompted to reflect on who, besides themselves, were responsible for their successes in life. Having a sense of gratitude and connectedness with others is critical to enhancing a person’s resilience and developing their wellbeing (Nelson-Coffey et al., 2019; Thompson, 2022). Two themes emerged: loved ones and having mentors.

It hasn’t been easy, I’ll be honest, but we’ve [them along with their spouse] managed to get through.

I have a bunch of mentors … she’s been a huge support. You choose your mentors based on the fact that you respect that person, and that they’re extremely knowledgeable.

The following participant explained how mentors can especially help in moments when the incident does not end well:

You have to find someone, especially nowadays, you have to find that mentor to kind of guide you through some of the stuff you’re going through. He was really good because he even told me right up, ‘Hey, man, you’re not going to win them all. You need to really be prepared for that.’ That’s kind of the thing that really helped me when we had a couple ones that didn’t go like we thought they’d go.

Previous awe literature has explained how awe experiences can be monumental, or once in a life time moments, such as the birth of a child or a visit to the Great Barrier Reef. Yet, awe experiences can also be found in moments we perceive as ordinary, everyday events and interactions (Schneider, 2009; Graziosi and Yaden, 2019; Shiota, 2021; Thompson, 2022a). Graziosi and Yaden (2019) and later Thompson (2022a), refer to this experience of awe as an expected and ordinary response to something extraordinary, or it can be an extraordinary response to something ordinary. A participant reflected on this notion of how awe can be experienced by a change in perspective:

I think it’s more about taking the focus off of yourself and then experiencing everything that was there, but you just didn’t see it. So, it’s almost like the glasses were off, things were out of focus and then, all of a sudden things become crystal clear … all of it.

And then you just start to impact different things around you differently …

If you can live that way, you’re far better off finding joy in the simplest things. Because you’re always going to find those simple things.

Self-care is an important element to further developing one’s mental health and personal resilience and was explored with the participants. Self-care is a multifaceted construct which, according to Martinez et al. (2021) involves the ability to care for oneself through “awareness, self-control, and self-reliance in order to achieve, maintain, or promote optimal health and well-being” (p. 423). Participants shared various practices they engage in and many explained how they purposely make time for self-care, which includes daily moments of awe:

Nature is my awe. As things get green and fill out here… I thrive on that, and we’ve had a long, long, cold winter, so when we can get back outside and feel the dirt and be in our yards, I think that’s my awe.

I think finding the ability to prioritize self-care, has been a really important aspect for me personally.

Having a diverse collection of self-care and resilience practices is important (Bonanno, 2013; Thompson et al., 2022) as the above participant continued:

I definitely exercise… I think the other thing that I find enjoyment is in social connectedness… I think [self-care] comes in different forms for different people, but I enjoy reading and listening to podcasts.

This participant further supported how valuable, social connectedness and personal relationships are with respect to self-care:

I have hobbies. I’m a world-traveler. Relationships are key, good friends and if you marry the right person, it’s going to help a lot.

Multiple participants expressed the importance of work-life separation being a valuable part of their self-care:

You have to separate from the job and you hopefully spend time with people other than those in your profession.

The following negotiator reflected on how their self-care has evolved over time, and it purposely includes having plants in their office:

Ten years ago, I’m not having plants in here. I’m just, I’m not having any plants in here … They’re not coming in here.

[Now] it just makes me feel good. And I have people come in here and I ask, ‘Do you feel relaxed?’ And they say, ‘Yeah. How come?’

I said, “Plants … They take the edge off.

Additionally, the above negotiator’s comment shows how their self-care, and resilience practice is also related to nature, which is a common category known for eliciting awe.

### Reflecting on awe as a resilience practice

In addition to collecting qualitative data to understand the role awe can have on the work of law enforcement hostage negotiators, the semi-structured interviews were also designed to be a resilience practice and intervention to enhance personal wellbeing. Broadly, sharing narratives can be supportive of an individual’s wellbeing (Frattaroli, 2006; Adler et al., 2016; Rutledge, 2016). Further, previous awe studies collected narratives from participants (Shiota et al., 2007; Campos et al., 2013; Piff et al., 2015; Cuzzolino, 2021) while other work suggests that sharing awe narratives as well as reflecting on moments of awe both in a person’s personal life and with work can enhance resilience in part, due to its relation to other practices (Thompson, 2022c). Thompson et al. (2022) explore the relationship between awe and other resilience practices such as cognitive reappraisal, gratitude, connectedness (with nature and others), prospection and optimism, and finding meaning and purpose in life and argue that these practices can support negotiators with their work as well as in their personal lives.

As previously mentioned, this exploratory study was designed in part to be a resilience practice for the participants. Based on the structuring of the interview questions, reflecting on awe moments can be related to other resilience practices such as cognitive reappraisal, social connectedness, gratitude, and others. Previous research has explored how awe-reflecting interviews can be supportive of one’s mental health while suggesting future research should follow up with interviewees to see if their participation impacted them in any way (Cuzzolino, 2021). The current study embraced that suggestion by asking during the initial interview if they had thought about awe in relation to their work prior to the interview, and regardless of their answer, what their thoughts are now that the interview was nearly over.

Multiple participants expressed that although they never connected the concept of awe with their work, they found it to be beneficial, especially in terms of reflecting:

No, I hadn’t. I hadn’t thought about awe, and I think it’s a term that I don’t… Yeah. I didn’t use quite frequently, but now I will find myself probably paying attention to those moments that I have, just an internal reflection on, I think that’s awe. Yeah, so thank you for that.

I haven’t thought about it too much other than what this morning’s conversation was with you … I’ll definitely reflect on this and think about it and maybe it’ll make me more positive and try and find something positive every day.

Continuing with the self-transcendent theme, the following participant wondered how it could be further shared with other negotiators:

It’s a really, really interesting concept, because I would never have put awe in negotiating… I just wonder if this could be incorporated into training more or a recognition amongst us.

This participant expanded on the above comments by also relating the self-transcendent impact awe can have by wanting to use future negotiator-related awe moments to be part of the team’s debriefing because now those incidents have a label, awe, to describe them:

No, not at all [having previously associated awe with law enforcement negotiation work]. You know what? I will share it with the rest of my guys, I will say, ‘Hey, remember those calls that I kept saying, the out of the ordinary ones? Now, we have a name for them. If you guys have an awe call, come and see me, and I’ll explain it.’

… Am I going to remember my awe moments? Yes. I’m going to put that name to it now.

Finally, this participant reflected on how associating awe with their work can enable a negotiator to discern the significance and impact of their work.

No. No, I haven’t. It’s when you really think about it, and you’re forced to reflect on specific incidents, then you’re like, ‘Wow, there really were a lot.’ But before this, it’s just, “Oh, they were a bunch of jobs I answered.’

This type of reflection can also be considered part of another resilience practice, finding meaning and purpose in life (Southwick and Charney, 2018; Thompson et al., 2022).

### Post-interview survey

This study further advanced the recommendations made by Cuzzolino (2021) by sending a brief, post-interview survey to each participant approximately five days after their interview was conducted. The purpose was to solicit their feedback on having participated in the study, and the impact it may or may not have had on them.

This study also utilized the suggestions of Thompson (2022c) that sharing awe narratives can be viewed as a resilience enhancing and wellness practice. Among the eight-question survey, participants were asked to briefly describe what it was like to be part of the study on awe to further examine the guiding research question:

I think it was helpful to talk about my experience as a negotiator, because it’s not something I have really allowed myself to do.

I enjoyed thinking about awe. It’s not a word I use very often, so I think taking the time to be very thoughtful about this particular question was enjoyable.

It was great having real discussions about awe in the world of negotiation.

This participant explained how cognitive reappraisal emerged from participating and enabling them to take time to reflect on their positive experiences:

By the end of the interview, I reflected on more positive jobs I have had in my career. Previously I would focus on the negative or heavy jobs. Thinking about awe and the positive is changing my outlook on things.

A subsequent question asked if there was anything specific they enjoyed about participating in the interview and many answers were similar to the previous question, especially with how the interview was conducted:

I enjoy being part of a discussion like this to open up my mind to different ideas.

It was an easy process and an open conversation.

Really enjoyable. Good conversation and very thoughtful.

Additionally, these participants explained how the interview allowed them to be pushed out of their comfort zone. Previous awe research has shown that experiencing awe can help people handle uncertainty (Shiota et al., 2006; Bonner and Friedman, 2011) and support law enforcement negotiators with their work (Thompson et al., 2022):

Questions were asked in a way that gave me the freedom to answer in a way that I thought was best. [The questions] also forced me to think of things that I hadn’t considered before.

The ability to think about an aspect of it that I had not really considered before.

After some time had passed, participants were asked if they had thought about awe. Awe has been described as a self-transcendent experience which motivates the person experiencing awe to want to help other. This participant explained how they have shared their experience of awe with others:

I have [thought about awe]. On more than one occasion I have chosen to verbalize my awe with a person. I think I have typically considered this a compliment, but in reality, awe is deeper than that. For my good friend it was related to her determination while attending school with two very young children. And secondly my oldest son who has received a few compliments lately from friends and I’ve also seen firsthand just how sensitive, loving and understanding he is with young children. The reason I believe this is an awe moment is that I have not observed many young boys with that type of demeanor.

The above quote can also demonstrate how experiences of awe are related to other resilience practices and attributes of experiencing awe: connectedness, and appreciating the accomplishments of others.

Participants also demonstrated the self-transcendent element of awe, as well as connectedness, by discussing their experiences from this study with other people and specifically, the concept of awe in relation to law enforcement negotiation:

Yes, I will be discussing it with my learner groups.

I mentioned this study to a few co-workers. No one had ever heard of the awe concept so it made for some interesting conversations.

Participants also linked the study and their participation directly with their negotiation work going forward:

After reflecting on it I can see how [awe] will be a valuable study for negotiators and preparing them for incidents that they may encounter.

How to recognize [awe] and deal with it in other negotiators.

Yes, how I can implement it into my next training session.

Yes! After the interview I attended a negotiators training day and shared the content of the interview with the team so we discussed awe. Also discussed it with a work colleague.

The final question asked participants if their perception of awe has changed since being interviewed for the study. This question is significant, as previous awe research has explained experiencing awe can identify gaps in one’s knowledge and importantly, motivate their curiosity to learn:

I think once I had an opportunity to think about awe, my perception surrounding recognition changed. I will notice my “awe” moments more now.

I do think it has in regards to being more recognizable. The concept was there but I had not really thought about it as far as how we discussed it. After doing this study I think it brought light on the importance of recognizing how awe plays into the majority of callouts we respond to.

As previously stated in other sections of this paper and consistent with previous research, the above comments suggest that the interviews contributed to a positive shift in participants’ perspectives going forward.

## Conclusion

Given the critical work law enforcement crisis and hostage negotiators are involved in, empirical research is necessary to continually examine their effectiveness, their ability to properly use the requisite skills, and their personal wellbeing. The relationship between the personal wellbeing of a negotiator and their ability to effectively engage in their work is closely intertwined. This exploratory study examined that relationship through a specific element of resilience – awe – and demonstrated how it can both support their wellbeing as well as their specialized police work. Through an analysis utilizing qualitative research, and the direct experiences of the participating negotiators, multiple themes emerged. These themes advance the previous awe research and uniquely identified how CHNs perceived awe and enhanced their role as negotiators.

This study offered a unique exploration into the phenomenon of awe and its relationship with CHNs. Importantly, the qualitative approach of this study was necessary when exploring a phenomenon such as awe, in order to understand the first-hand perspective of the CHNs. This is an exploratory study and while it is important to gain this first-hand perspective, a limitation of this study is that it focuses on a single methodological approach to examine awe and negotiators. Future research can advance this study in numerous ways. Additional studies can include utilizing quantitative methodologies with larger samples to increase the diversity of responses and conducting longitudinal studies to examine how awe can potentially support negotiators’ skills, practice, and personal wellbeing over a period of time. Considering this study is the first to explore CHN’s experiences and awe, there is significant room for future research that embraces diverse methodologies. By exploring the positive effect of awe reflection and providing an overview of various themes, this study has established the groundwork for additional analyses related to awe and CHNs.

## Data availability statement

The raw data supporting the conclusions of this article will be made available by the authors, without undue reservation.

## Ethics statement

The studies involving human participants were reviewed and approved by Lipscomb University. The patients/participants provided their written informed consent to participate in this study.

## Author contributions

All authors listed have made a substantial, direct, and intellectual contribution to the work and approved it for publication.

## Conflict of interest

The authors declare that the research was conducted in the absence of any commercial or financial relationships that could be construed as a potential conflict of interest.

## Publisher’s note

All claims expressed in this article are solely those of the authors and do not necessarily represent those of their affiliated organizations, or those of the publisher, the editors and the reviewers. Any product that may be evaluated in this article, or claim that may be made by its manufacturer, is not guaranteed or endorsed by the publisher.

## Footnotes

^1^For a more in-depth review of CHN skills, see Thompson et al. (2022).

^2^For example, see the review provided by Thompson et al. (2022).

## Appendix

### Guiding questions for hostage negotiators (Thompson and Jensen, 2023)

1. I’d like to start by learning a little about the path that led you to become a hostage negotiator. What drew you to this field and your current position?
2. What’s the most important part of your job? What makes someone good at being a negotiator (including team concept)?
3. How much did you want to be a negotiator?
4. What is the most meaningful thing that has happened to you so far while working as a hostage negotiator?
  Who else was part of it?
5. Who else has been responsible for your life’s successes?
6. What is your definition of awe?
7. How would you describe what it is like to experience an awe moment? Be descriptive as possible (not giving an example though). What level of intensity were those emotions?
8. Have you ever experienced awe in the context of your hostage negotiation work? Can you tell me about it? Did this experience change your thinking in any way? How about your behavior?
9. Can you recall any other awe experiences in the context of your work?
10. Can you share a non-work-related awe experience?
11. Would you describe those awe experiences you shared as isolated or unusual incidents, or the feeling of awe is something you experience with some regularity at work or outside of it?
12. What role, if any, does experiencing awe have with respect to your work as a hostage negotiator?
13. Some people describe awe as an experience of feeling part of something larger than yourself. Does that resonate with you, and with your experiences?
14. Sometimes when someone experiences awe, it creates what is described as a perspective shift or change. What are your thoughts on this, based on your experiences?
15. What are some daily moments that you try to enjoy and possibly find awe in?
16. Have you ever thought about awe before in the context of your work? What is it like now?
17. Now that this interview is nearly over, has it raised any other thoughts or ideas that you’d like to share?
18. Do you have any questions for me?
19. What self-care practices do you do that can be related to awe?
